# Supplementary material for: Variation in female reproductive tract morphology across the reproductive cycle in the zebra finch
Source: PeerJ. 2020 Nov 11;8:e10195. doi: 10.7717/peerj.10195 (PMC7666545; doi:10.7717/peerj.10195)
Supplement: Supplemental Information 2 [file peerj-08-10195-s002.pdf]

R-Code for Variation in female reproductive tract morphology and estradiol across the reproductive cycle in the zebra finch

Laura L. Hurley*, Ondi L. Crino, Melissah Rowe, Simon C. Griffith

Generated 15/06/2020 - Original finalized 26/03/2020

Base libraries (others in code)

```
#R version in use: 3.5.3

library(lme4)
library(lmerTest)
library(tidyr)
library(doBy)
library(emmeans)
library(piecewiseSEM)
library(yarrr)
SE <-function(x)sd(x)/sqrt(length(x))
```

Data and functions

```
euthFemale <- read.csv("~/FemaleReproTrack.csv", stringsAsFactors = TRUE, hea
der = TRUE)

euthOvaduct <- euthFemale %>% drop_na(OvaductWT)
euthFol <- euthFemale %>% drop_na(Follicle_Vol)
euthE2 <- euthFemale %>% drop_na(E2_2_NA)

overdisp_fun <- function(model) {
    rdf <- df.residual(model)
    rp <- residuals(model,type="pearson")
    Pearson.chisq <- sum(rp^2)
    prat <- Pearson.chisq/rdf
    pval <- pchisq(Pearson.chisq, df=rdf, lower.tail=FALSE)
    c(chisq=Pearson.chisq,ratio=prat,rdf=rdf,p=pval)
}

#Calculating Scaled Mass index - This column is already saved into the data f
ile
#http://apansharing.blogspot.com/2018/05/an-r-function-olsrobust-caled-mass-i
ndex.html
scaledMassIndex <-
  function(x, y, x.0 = mean(x)) {
    require(smatr)
    require(magrittr)
    require(MASS)
    require(data.table)
```

```
    logM.ols <- lm(log(y) ~ log(x))
    logM.rob <- rlm(log(y) ~ log(x), method = "M")
    b.msa.ols <- coef(sma(log(y) ~ log(x)))[2]
    b.msa.rob <- coef(sma(log(y) ~ log(x), robust = T))[2]
    SMI.ols <- y * (x.0 / x) ^ b.msa.ols
    SMI.rob <- y * (x.0 / x) ^ b.msa.rob
    #res <- data.frame(SMI.ols, SMI.rob, x, y)
   # pred.DT <-
    #  data.table(x = seq(min(x), max(x), length = 100)) %>%
     # .[, y.ols := predict(logM.ols, newdata = .) %>% exp] %>%
      #.[, y.rob := predict(logM.rob, newdata = .) %>% exp]
    #attr(res, "b.msa") <- c(ols = b.msa.ols, rob = b.msa.rob)
   # return(res)
  }


euthFemale$dt.SMIF <-  scaledMassIndex(euthFemale$`Tarsus_female`, euthFemale
$`Mass_female`, x.0 = 2) #This is already saved into the data file as SMI_F
```

Female SMI

```
FSMI0 <- lm(SMI_F ~TimePt, data=euthFemale)
summary(FSMI0)
anova(FSMI0)

overdisp_fun(FSMI0)
```

Oviduct Wet Mass

```
euthOvaduct$OvaductWTln <- log(euthOvaduct$OvaductWT)

Ovaduct0 <- lm(OvaductWTln~TimePt+SMI_F, data=euthOvaduct)
summary(Ovaduct0)
anova(Ovaduct0) #pre is sig diff D3, mid, 6dph, fledge

overdisp_fun(Ovaduct0)

emmeans(Ovaduct0, pairwise~TimePt)

Ovar <- resid(Ovaduct0)
op <- par(mfrow = c(2, 2), mar = c(5, 4, 1, 2))
plot(Ovar)
qqnorm(Ovar); qqline(Ovar, col = 2)
hist(Ovar, xlab = "Ovaduct Residuals", main = "")
plot(euthOvaduct$TimePt, Ovar, xlab = "TimePt", ylab = "Ovaduct Residuals")

par(op)
#normality of residuals
shapiro.test(Ovar)
#Bartlett test of homogeneity of variance
```

```
bartlett.test(Ovar,euthOvaduct$TimePt)

rsquared(Ovaduct0)
```

Follicle Volume

```
euthFol$Follicle_Volln <- log(euthFol$Follicle_Vol)

Fol0 <- lm(Follicle_Volln~TimePt+SMI_F, data=euthFol)
summary(Fol0)
anova(Fol0) #pre is sig diff  mid, 6dph, fledge

overdisp_fun(Fol0)

emmeans(Fol0, pairwise~TimePt)

Folr <- resid(Fol0)
op <- par(mfrow = c(2, 2), mar = c(5, 4, 1, 2))
plot(Folr)
qqnorm(Folr); qqline(Folr, col = 2)
hist(Folr, xlab = "Ovaduct Residuals", main = "")
plot(euthFol$TimePt, Folr, xlab = "TimePt", ylab = "Ovaduct Residuals")

par(op)
#normality of residuals
shapiro.test(Folr)
#Bartlett test of homogeneity of variance
bartlett.test(Folr,euthFol$TimePt)

rsquared(Fol0)
```

Yolky Follicle number

```
#This results in the CIS for yolks
library(loo) # required to use function compare()
library(processx) #required to load rstranarm with newer resions of R
library(rstanarm)

# compare fitted models
m1 <- stan_glm(data = euthFol, Yolky ~ TimePt-1, family = neg_binomial_2) # d
rop intercept using "-1" so each coefficient corresponds to a mean count at e
ach time period
summary(m1)
m2 <- stan_glm(data = euthFol, Yolky ~ 1, family = neg_binomial_2)
compare(loo(m1),loo(m2))
pnorm(-14.5/2.7) # approximate p value

# posterior interval (compare to evaluate significance) - exp() of the values
to get counts on the arithmetic scale
M3 <- exp(posterior_interval(m1,prob=0.95))
```

```
summary(M3)
M3
```

Construction of Figure 3

```
x0 <-c(1:6)
xname0 <- c("Pre-breed", "Nesting", "Laying", "Incubation", "Nestling", "Fled
ge")

dev.off()
#par(mar=c(7,6,3,3), cex.axis=1)
Fig3 <- {
  par(mfrow = c(3,1))
par(mar=c(1,5,1,3), cex.axis=1)
pirateplot(formula = OvaductWT ~ TimePt,
           data = euthOvaduct,
           theme = 0,
           xlab = "",
           xaxt = "n", #remove pirate plots auto xaxis
           ylab = "Oviduct Mass (g)",
           main = NULL,
           pal = "black", #set palette to black and white
           inf.method = "se",
           bean.b.o = .6, # Bean boarder - bean.f.o would fill colour
           point.o = .7, # Points
           jitter.val= 0.08, #shifts points
           inf.f.o = .7, # Inference fill
           inf.b.o = .8, # Inference border
           avg.line.o = 1, # Average line
           bar.f.o = .5, # Bar
           inf.f.col = c("grey85"), # Inf fill col
           inf.b.col = "black", # Inf border col
           avg.line.col = "black", # avg line col
           bar.f.col = gray(1), # bar filling color,
           gl.lwd = c(0, 0),
           point.pch = 20,
           point.cex = 2)
axis(1, at=c(1:6), labels=F, las=2)


#par(op)

#dev.off()
#par(mar=c(7,6,3,3), cex.axis=1)
par(mar=c(1,5,1,3), cex.axis=1)
pirateplot(formula = Follicle_Vol ~ TimePt,
           data = euthFol,
           theme = 0,
           yaxt = "n",
           xlab = "",
```

```
           xaxt = "n", #remove pirate plots auto xaxis
           ylab = "Follicle Volume"~(mm^{3}),
           main = NULL,
           pal = "black", #set palette to black and white
           inf.method = "se",
           bean.b.o = .6, # Bean boarder - bean.f.o would fill colour
           point.o = .7, # Points
           jitter.val= 0.12, #shifts points
           inf.f.o = .7, # Inference fill
           inf.b.o = .8, # Inference border
           avg.line.o = 1, # Average line
           bar.f.o = .5, # Bar
           inf.f.col = c("grey73"), # Inf fill col
           inf.b.col = "black", # Inf border col
           avg.line.col = "black", # avg line col
           bar.f.col = gray(1), # bar filling color
           gl.lwd = c(0, 0),
           point.pch = 20,
           point.bg = "white",
           point.col = "black",
           point.cex = 2)
axis(1, at=c(1:6), labels = F, las=2)
axis(2, at=seq(0, 200, by = 50), las=1)

par(mar=c(6,5,1,3), cex.axis=1)
pirateplot(formula = Yolky ~ TimePt,
           data = euthFol,
           theme = 0,
           yaxt = "n",
           xlab = "",
           xaxt = "n", #remove pirate plots auto xaxis
           ylab = "Number of Preovulatory Follicles",
           main = NULL,
           pal = "black", #set palette to black and white
           inf.method = "se",
           bean.b.o = .6, # Bean boarder - bean.f.o would fill colour
           point.o = .7, # Points
           jitter.val= 0.09, #shifts points
           inf.f.o = .7, # Inference fill
           inf.b.o = .8, # Inference border
           avg.line.o = 1, # Average line
           bar.f.o = .5, # Bar
           inf.f.col = c("grey73"), # Inf fill col
           inf.b.col = "black", # Inf border col
           avg.line.col = "black", # avg line col
           bar.f.col = gray(1), # bar filling color,
           gl.lwd = c(0, 0),
           point.pch = 20,
           point.bg = "white",
           point.col = "black",
```

```
           point.cex = 2)
axis(1, at=c(1:6), labels = xname0, las=2)
axis(2, at = seq(from = 0, to = 6, by = 1), las=1)
}
```
