# Supplementary material for: Variation in female reproductive tract morphology across the reproductive cycle in the zebra finch
Source: PeerJ. 2020 Nov 11;8:e10195. doi: 10.7717/peerj.10195 (PMC7666545; doi:10.7717/peerj.10195)
Supplement: Supplemental Information 3 — Change in reproductive tissue in seasonal breeders and change in Zebra finch from this and previous studies (below double hatched line). Note only studies reporting actual quantitatively measured oviduct mass and/or follicle volume, are used to make comparison to the current study. Other studies do exist that use qualitative scoring of development/condition. [file peerj-08-10195-s003.docx]

**Variation in female reproductive tract morphology across the reproductive cycle in the zebra finch**

Laura L. Hurley, Ondi L. Crino, Melissah Rowe, Simon C. Griffith

**Table S1. Variation in gonadal development in female passerine birds.** Change in reproductive tissue in seasonal breeders and change in Zebra finch from this and previous studies (below double hatched line). Note only studies reporting actual quantitatively measured oviduct mass and/or follicle volume, are used to make comparison to the current study. Other studies do exist that use qualitative scoring of development/condition.

| **Species** | **Tissue (measure)** | **min** | **max** | **Fold increase** | **Sampling Point (min/max)** | **Conditions/ comments** | **Reference** |
| --- | --- | --- | --- | --- | --- | --- | --- |
| House sparrow  *Passer domesticus* | Oviduct mass (mg) | 3.3 | 241.1 | 73.1 | Dec/March | Wild, free-living birds, | Keck, 1934 |
|  | Oviduct mass (mg) | 0.006 | 0.333 | 55.5 | Late winter-early spring/ laying | Dry mass | Krementz and Ankney 1986 |
|  | Follicle size (mm)* | 0.1 | 10 | 100 | Postnuptial molt/laying | Largest follicle diameter | Hegner and Wingfield 1986 |
| European Starling  *Sturnus vulgaris* | Oviduct mass (mg) | 80 | 3990 | 49.9 | Non-reproductive/ Reproductive | Wet mass, | Vézina and Salvante, 2010 |
|  | Follicle size (mg) | 10 | 1190 | 119.0 | Non-reproductive/ Reproductive | Wet mass | Vézina and Salvante, 2010 |
| Clay-colored robin  *Turdus grayii* | Follicle size (mm)* | 0.5 | 4.7 | 9.4 | Pre-breed/ Peak -breed | Largest follicle diameter | Wikelski et al., 2003 |
| Blue-gray tanager  *Thraupis episcopus* | Follicle size (mm)* | 0.5 | 3.7 | 7.4 | Pre-breed/ Peak -breed | Largest follicle diameter | Wikelski et al., 2003 |
| Song wren  *Cyphorhinus phaeocephalic* | Follicle size (mm)* | 0.5 | 2.3 | 4.6 | Pre-breed/ Peak -breed | Largest follicle diameter | Wikelski et al., 2003 |
| Great tit  *Parus major* | Oviduct mass (mg)* | 1 | 220 | 220 | Non-breeding (late Sept-mid-March)/ Pre-lay (late April) | Wet, fixed tissue. | Silverin 1978 |
| Pied flycatcher  *Ficedula hypoleuca* | Oviduct mass (mg) | 180 | 1000 | 5.6 | Laying/ Incubating | Wet mass. | Ojanen, 1983 |
| Willow tit  *Poecile montanus* | Follicle size (mm)* | 0.25 | 3.84 | 15.4 | 14d at 20 hrs light/57d at 20 hrs light | Largest follicle diameter. Paired females** | Silverin and Westin 1995 |
| Pied myna  *Sturnus contra contra* | Oviduct mass (mg/100g body weight) | 23.6 | 930.2 | 39.4 | Nov/May | Based on monthly measures across year | Gupta and Maiti 1987 |
|  |  | 122.1 | 2350.4 | 19.2 | Early nest/ Egg laying | With in nesting cycle | Gupta and Maiti 1987 |
| White-crowned sparrow  *Zonotricha leucophrys* | Follicle size (mm)* | < 0.5 | >5 | ≥10 | Winter flock/Laying | Category, not actual measure. | Wingfield and Farner, 1978 |
|  | Follicle size (mm) | 0.77 | 6.6 | 8.6 | Non-breeding/ Laying | Free-living | Kern, 1972 |
|  | Follicle size (mm) | 0.4 | 1.8 | 4.5 | Non-breeding/ Laying | Captive in outdoor aviaries | Kern, 1972 |
| Western Meadowlark  *Sturnella neglecta* | Follicle size (mm)* | 0.75 | 26 | 34.7 | Post-breed (early July)/ Breeding (mid-May) | Largest follicle diameter | Wingfield and Farner, 1993 |
|  |  |  |  |  |  |  |  |
| Zebra Finch  *Taeniopygia guttata* | Follicle size (mm^3^) | 2.45 | 74.9 | 30.6 | Post-hatch/ Nesting | With-in single breeding event | Current study |
|  | Oviduct mass (g) | 0.03 | 0.43 | 14.3 | Post-hatch/ Laying |  | Current study |
|  | Follicle size (mm^3^)* | 3 | 44 | 14.7 | Non-breed/ Early-breed | Predictable environment^+^ | Perfito et al., 2007 |
|  | Follicle size (mm^3^)* | 5 | -- | -- | Non-breed | Unpredictable environment^+^ | Perfito et al., 2007 |
|  | Follicle size (mm) | 1.8 | 3.9 | 2.2 | Water restricted/ ad lib water^++^ |  | Prior et al., 2013 |
|  | Oviduct mass (g) | 0.07 | 0.23 | 3.2 |  |  | Prior et al., 2013 |

* Approximated from figure, ** experimental study, may not be fully developed, + breeding stage inferred, ++ experimental study, may not be fully developed or regressed, with breeding controlled via egg removal.

**References:**

Gupta S, and Maiti B. 1987. Seasonal changes in the oviduct of the pied myna (Aves: Sturnidae). *Journal of morphology* 194:247-263.

Hegner RE, and Wingfield JC. 1986. Behavioral and endocrine correlates of multiple brooding in the semicolonial house sparrow *Passer domesticus* II. Females. *Hormones and Behavior* 20:313-326.

Keck WN. 1934. The control of the secondary sex characters in the English sparrow, *Passer domesticus* (Linnaeus). *Journal of Experimental Zoology* 67:315-347.

Kern MD. 1972. Seasonal changes in the reproductive system of the female white-crowned sparrow, *Zonotrichia leucophrys gambelii*, in captivity and in the field. *Zeitschrift für Zellforschung und Mikroskopische Anatomie* 126:297-319.

Krementz DG, and Ankney CD. 1986. Bioenergetics of egg production by female house sparrows. *The Auk* 103:299-305.

Ojanen M. 1983. Egg development and the related nutrient reserve depletion in the pied flycatcher, *Ficedula hypoleuca*. Annales Zoologici Fennici: JSTOR. p 293-299.

Perfito N, Zann RA, Bentley GE, and Hau M. 2007. Opportunism at work: Habitat predictability affects reproductive readiness in free-living zebra finches. *Functional Ecology* 21:291-301.

Prior NH, Heimovics SA, and Soma KK. 2013. Effects of water restriction on reproductive physiology and affiliative behavior in an opportunistically-breeding and monogamous songbird, the zebra finch. *Hormones and Behavior* 63:462-474.

Silverin B. 1978. Circannual rhythms in gonads and endocrine organs of the great tit *Parus major* in south-west Sweden. *Ornis Scandinavica*:207-213.

Silverin B, and Westin J. 1995. Influence of the opposite sex on photoperiodically induced LH and gonadal cycles in the willow tit (*Parus montanus*). *Hormones and Behavior* 29:207-215.

Vézina F, and Salvante KG. 2010. Behavioral and physiological flexibility are used by birds to manage energy and support investment in the early stages of reproduction. *Current Zoology* 56:767-792.

Wikelski M, Hau M, Douglas Robinson W, and Wingfield JC. 2003. Reproductive seasonality of seven neotropical passerine species. *The Condor* 105:683-695.

Wingfield J, and Farner DS. 1993. Endocrinology of reproduction in wild species (In: Avian Biology, vol. IX, Eds: DS Farner, JR King, KC Parkes)–Academic Press. *New York*:163-327.

Wingfield JC, and Farner DS. 1978. The endocrinology of a natural breeding population of the white-crowned sparrow (*Zonotrichia leucophrys pugetensis*). *Physiological Zoology* 51:188-205.
